# Weight Loss Maintenance: Have We Missed the Brain?

**DOI:** 10.3390/brainsci8090174

**Published:** 2018-09-11

**Authors:** Dimitrios Poulimeneas, Mary Yannakoulia, Costas A. Anastasiou, Nikolaos Scarmeas

**Affiliations:** 1 Department of Nutrition and Dietetics, Harokopio University, GR 17676 Athens, Greece; dpoul@hua.gr (D.P.); myiannak@hua.gr (M.Y.); acostas@hua.gr (C.A.A.); 2 Eginition Hospital, 1st Neurology Clinic, Department of Social Medicine, Psychiatry and Neurology, National and Kapodistrian University of Athens, GR 15772 Athens, Greece; 3 Taub Institute for Research in Alzheimer’s Disease and the Aging Brain, The Gertrude H. Sergievsky Center, Department of Neurology, Columbia University, New York, NY 10027, USA

**Keywords:** obesity, weight loss maintenance, maintainers, regainers, neural processing, functional neuroimaging

## Abstract

Even though obese individuals often succeed with weight loss, long-term weight loss maintenance remains elusive. Dietary, lifestyle and psychosocial correlates of weight loss maintenance have been researched, yet the nature of maintenance is still poorly understood. Studying the neural processing of weight loss maintainers may provide a much-needed insight towards sustained obesity management. In this narrative review, we evaluate and critically discuss available evidence regarding the food-related neural responses of weight loss maintainers, as opposed to those of obese or lean persons. While research is still ongoing, available data indicate that following weight loss, maintainers exhibit persistent reward related feeling over food, similar to that of obese persons. However, unlike in obese persons, in maintainers, reward-related brain activity appears to be counteracted by subsequently heightened inhibition. These findings suggest that post-dieting, maintainers acquire a certain level of cognitive control which possibly protects them from weight regaining. The prefrontal cortex, as well as the limbic system, encompass key regions of interest for weight loss maintenance, and their contributions to long term successful weight loss should be further explored. Future possibilities and supportive theories are discussed.

## 1. Introduction

Obesity remains a major public health concern at the global level. Excess body weight has been associated with negative effects in multiple organs and body systems [1,2,3], including the peripheral and the central nervous system. Being obese is associated with several neuropathologies [4], ranging from polyneuropathy [5] and impaired function of various cognitive domains [6] to neurodegenerative diseases, like Alzheimer’s disease [7] and other dementias [8,9]. The introduction of neuroimaging in obesity management has further yielded useful information [10]. Obese individuals have been known to exhibit hypothalamic abnormalities [11] hippocampal atrophy [12], and lower brain volume compared to normal-weight or overweight controls [13]. Obesity is associated with both structural and functional alterations in brain areas related to reward anticipation [14,15], inhibition and restraint [16], as well as higher cognitive functioning [17].

On the other hand, weight loss has been found to mitigate neurodegeneration and cognitive decline. In a recent meta-analysis, even modest weight loss (≥2 kg) was associated with improvements in attention, memory, executive functioning and language [18]; larger losses (>10% of initial body weight) have been found to augment cognition in the elderly [19]. The weight loss method may also be of neurological importance. For instance, when compared to behavioral dieters, patients following bariatric surgery have shown enhanced processing in areas related to food motivation (bilateral temporal cortex) [20]. In the same study, dieters showed increased hunger (right medial prefrontal cortex, left precuneus) and self-referent processing, when compared to bariatric patients [20].

However, weight loss is not a milestone, but rather part of a dynamic process. Following weight loss, the weight-reduced individual enters an uneven combat, commonly resulting inweight regain. The available data indicate that the trend of weight regaining in dieters is highly eminent [21] and that ex-obese persons maintain a mere 3–4 kg of their initial weight loss [22], or even less [23]. Hence, researchers have recently focused their interest on individuals who experienced long-term successful weight loss maintenance (SWLM). Several weight control registries have been established worldwide, including the National Weight Control Registry in the US and the MedWeight study in Greece [24,25,26,27]. These registries have delineated several factors involved in SWLM, including dietary behaviors [28,29,30,31], lifestyle habits [32,33,34,35,36] and psychosocial aspects [37,38,39], that assist individuals in the longevity of their weight loss.

Despite this knowledge, existing models explain only 30% or less of maintenance variance, leaving the rest unexplained and unexplored [40], suggesting that the nature of maintenance is yet poorly understood. Along these lines, little is known on the sustainability of brain changes during weight loss maintenance. Thus, some research interest has been focused on the neural mechanisms that are involved in weight management and how they could potentiate long term success in post-dieters. In this narrative review, we present and critically discuss weight loss maintenance in relation to the neural processing of individuals with a history of obesity compared to that of lean and/or obese counterparts.

## 2. Materials and Methods

**Figure 1:**
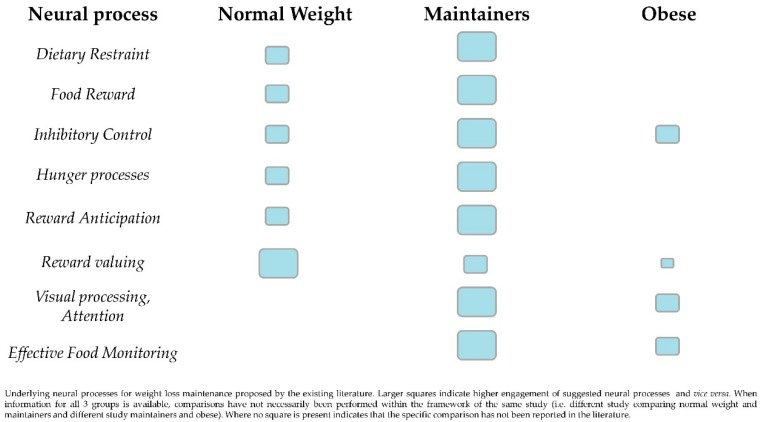
A summary of regional brain activity and proposed function in maintainers compared to obese and normal weight individuals.

We searched PubMed for functional neuroimaging studies. Combinations of the following keywords were used: weight loss maintenance/maintainer, weight regain/regainer, functional magnetic resonance imaging (fMRI) or positron emission tomography (PET), neural activity/processing. In addition, the references of the retrieved studies were searched for similar research. Inclusion criteria for this narrative review were (i) publication date from January 2000 till May 2018, (ii) investigations involving human subjects >18 years of age, with absence of psychopathology, (iii) involvement of weight loss maintainers and/or regainers in the sampling. Articles that were involving animal studies, basic neurobiological research, or did not meet the inclusion criteria, were excluded. Our search concluded in 8 studies, and their descriptive information can be found in Table 1. Regional brain activation differences among maintainers, obese and normal weight individuals are summarized in Figure 1.

## 3. The Neural Background of Weight Loss Maintenance

The notion that cognitive skills may be important for SWLM has interested obesity researchersover the previous decades. In a 2001 study, after a weight reduction intervention, provision of extended care with cognitive component (i.e., problem solving) resulted inmaintenance of >10% of weight loss [48]. What is more, this form of extended care appeared to be more effective for SWLM than relapse prevention training and no extended care. Few years later, Del Parigi and associates [41,42], in two PET-scan studies involving weight loss maintainers, obese and normal weight volunteers recorded that the posterior cingulate and the amygdala were activated after a satiating meal in the obese group, but not in the normal weight or maintainers. In the same study, persistent abnormal responses in the middle insular cortex and the hippocampus of maintainers and obese persons were also reported. These results were of the first to suggest that, being obese or having a history of obesity is associated with greater craving for the coming meal and enhanced memory processing. Additionally, these findings also suggest that when individuals succeed with weight loss, their brain activity differentiates to some extent in relation to their previous obese state.

## 4. The Interplay of Restraint and Reward Anticipation Brain Regions

As already stated, there seems to exist a strong link between obesity and impaired function of the reward network. The mechanisms could conceivably be explained by the reward-deficiency model [49]. Overweight and obese individuals exhibit greater activation in reward related areas (i.e., insula, amygdala, cingulated gyrus) and reward anticipation areas (anterior cingulate, orbitofrontal cortex) [50]. Higher BMI (Body Mass Index) has been associated with higher activation of reward anticipation and impulsivity regions (anterior cingulated cortex, middle frontal gyrus) in both cross-sectional and prospective studies [51,52]. Finally, a recent systematic review of functional neuroimaging studies suggests consistency in the published research that relates obesity with high reward-related region activation, even after the consumption of a high-calorie meal [50].

In studies involving weight loss maintainers, the picture is similar. However, reward-related processes appear to activate in parallel to a different set of brain areas. Following an orosensory paradigm, Sweet and colleagues [45] observed elevated responses in almost all brain regions examined in maintainers, compared to obese and normal weight controls. As only maintainers exhibited significant reactivity in the left putamen and inferior frontal gyrus (areas associated with food reward and inhibitory control, respectively), the authors hypothesized that maintainers exhibited greater reward expectations during the orosensory stimulation, but responded with greater restraint. Greater restraint in maintainers has been previously reported also in an observational study using visual stimuli of low and high calorie food pictures [43]. Compared to obese and normal weight controls, the maintainers seemed to experience greater inhibition and restraint, as they showed greater activation in the left superior frontal region of the brain, this “inhibitory” activation of the prefrontal cortex that maintainers experience is similar to the pattern found in normal-weight individuals [53,54].

Even though the activation of the inferior frontal gyrus during various stimuli has been associated with SWLM, the prospective study by Murdaugh et al. did not support it [46]: weight maintenance 9 months following a dietary intervention was associated with decreased post-treatment activity in the insula and the putamen, as well as the midbrain/thalamus and the inferior frontal gyrus. These contradictory findings may be partly explained by the different methodologies and selection criteria used in various studies.

According to Sweet et al. [45], when maintainers are provided with a food stimulus, their brain reaction follows a pattern of elevated reward expectation, yet consequently greater inhibitory control. In a prospective study, greater impulse control (as expressed by the activation of the dorsolateral prefrontal cortex), immediately after a 12-week behavioral weight loss intervention, was found to be predictive of SWLM in the 1-year follow up [55]. Additionally, a recent study exploring cortical thickness as a surrogate marker of cognitive control, concluded that maintainers tended to have greater cortical thickness than obese controls (although the trend did not pass the significance threshold) [44]. Taken together these findings may imply that, in the post-dieting period, maintainers could exhibit cognitive changes, which counteract their food reward-related neural circuits and possibly protect them against weight regain.

## 5. Executive Functions Driving Maintenance

As the prefrontal cortex may play a pivotal role in protecting against weight regaining the potential involvement of executive functioning has been investigated. Executive functions, a neuropsychological trait regulated by the prefrontal cortex [56], mediate processes of “how” choices are made and established [57] and may moderate the relationship between eating intention and behavior [58]. Higher executive functions have been linked to weight loss maintenance during the 2-year post-procedural period of bariatric surgery patients [59]. Similarly, in a functional neuroimaging study of people post obesity surgery, greater utilization of the executive control circuitry of resistance to palatable food cues was associated with more successful maintenance [51]. Thus, manipulation of executive functions through cognitive training, towards enhancing and optimizing function of the prefrontal cortex, may hold promising aspects for long term weight loss maintenance [60,61].

## 6. Clinical Implications and Future Possibilities

In summary, while evidence is still insufficient, following weight loss, maintainers acquire a certain level of cognition, reacting with heightened inhibition against food cues, to compensate for elevated reward related feeling over food. The differences in the neural activity of weight loss between maintainers and obese individuals indicate a persistent imbalance between hedonic and homeostatic food ingestion following weight loss. The prefrontal cortex, as well as the limbic system are key regions of interest for weight loss maintenance and their contributions to long term successful weight loss should be further explored.

Although some evidence regarding the neural responses of weight loss maintainers has been accumulated, there are major caveats in the comparison and reproducibility of the results from the neuroimaging studies [50]. As indicated in Table 1, the measures of acquisition have not been systematic, there is no standard approach in methodology (scan acquisition, inclusion criteria etc.) while the studies so far have commonly used small-sized, convenient samples. What is more, not all regions that activate differently in maintainers in comparison to obese or normal weight individuals have been assigned a proposed function, thus inadequately profiling neural involvement in weight loss maintenance (Figure 1). Future research should address these issues and exploit standardized approaches in larger population groups.

Following weight loss, maintainers exhibit a transitioning period during which they show brain similarities to both obese and normal weight persons. The duration of the transitioning period, if finite, is yet unknown: it begins after weight loss and may span beyond 3 years of maintenance. New studies should examine the brain regions of people who have achieved to maintain their weight loss for prolonged periods of time (i.e., ≥5 years of maintenance) that activate, when exposed to food cues. In addition, neuroimaging research should directly compare weight loss maintainers with individuals that regained their weight loss shortly after the dieting period, or maintained it for more than a year, but regained the loss thereafter (ex-maintainers).

The interplay of restraint and reward for weight loss maintenance could be explored from different perspectives. Multistep cognitive behavioral treatment in obese patients has produced promising results in enhancing dietary restraint [62]. Our understanding of neural restraint in weight loss maintenance would be enhanced by studies examining not only the neural activity of selected brain regions, but also their functional connectivity in the resting state. For example, resting state activity of the middle temporal gyrus has been shown to correlate with dietary restraint [63]; this association was supposed to reflect the middle temporal gurus’ connectivity with frontal regions involved in inhibitory processes [64]. Exploring the sensory experience of food, as well as the impact of food architecture may also be of importance in addressing food reward [65]. Personality traits, such as persistence [66], may favor weight loss maintenance, and their implications in the abovementioned interplay should be further researched. Last, examining the role of neuropeptides with known homeostatic properties, prominent in the limbic system and the prefrontal cortex, such as orexin [67,68], may withhold therapeutic targets for long-term obesity management [69].

To succeed with weight loss maintenance, post obese individuals are required to exercise morethan a dieter [70], and to adhere to a low-calorie diet [28], 300–400 kcal lower of that expected of their body mass [71], to compensate for decreased energy expenditure and persistent physiological adaptations that favor weight regain [72].Considering the brain similarities of the maintainers to both the obese and normal-weight persons, we hypothesize that people with previous history of obesity, even if presented with normal BMI, should not be treated as normal-weight, but rather as ex-obese or weight-reduced individuals. This postulation is strengthened by research that focuses beyond behavior or neuroimaging. For instance, epigenetic DNA methylation patterns of maintainers has been found to more closely resemble that of normal weight than obese controls [73]. If supported by future research, this hypothesis may provide a paradigm shift for clinicians and obesity researchers, so as to more thoroughly profile and prevent weight regaining.

## Figures and Tables

**Table 1:** Descriptive characteristics of the reviewed neuroimaging studies (*n =* 8).

Study, Study Design	Study Population	Weight Loss Maintenance Definition	Imaging Method	Exclusion Criteria	Measures
Del Parigi et al., 2004 [41] Observational Case-Control	11 Maintainers 23 OB 21 NW	Stable weight for ≥3 months, after intentional weight reduction from a BMI ≥ 35 kg/m^2^ to <25 kg/m^2^, through diet and exercise	PET-scan	Not reported	Regional cerebral blood flow at baseline (after a 36-h fast), after tasting and after consuming a satiating liquid meal, in 4 brain regions
Del Parigi et al., 2007 [42] Observational Case-Control	9 Maintainers 20 NW	Stable weight for ≥3 months, after intentional weight reduction from a BMI ≥ 35 kg/m^2^ to <25 kg/m^2^, through diet and exercise	PET-scan	Not reported	Brain response to the sensory experience of food and meal consumption
McCaffery et al., 2009 [43] Observational Case-Control	17 Maintainers 16 OB 18 NW	Maintenance of intentional weight loss ≥13.6 kg, from a maximum BMI ≥ 30 kg/m^2^ to normal BMI, for at least 3 years	fMRI	Medication Left-handedness Neuropathology Psychopathology Standard MRI contradictions	Visual stimuli of low and high calorie foods and non-foods, in a single 8-min run, after a 4-h fast
Hassenstab et al., 2012 [44] Observational Case-Control	17 Maintainers 17 OB 19 NW	Maintenance of intentional weight loss ≥13.6 kg, from a maximum BMI ≥ 30 kg/m^2^ to normal BMI, for at least 3 years	MRI	Medication Neuropathology Psychopathology Standard MRI contradictions	Cortical thickness in 4 *a-priori* set brain regions of the cognitive control network
Sweet et al., 2012 [45] Observational Case-Control	17 Maintainers 14 OB 18 NW	Maintenance of intentional weight loss ≥13.6 kg, from a maximum BMI ≥ 30 kg/m^2^ to a BMI≥18.5 and <27 kg/m^2^, for at least 3 years	fMRI	Medication Left-handedness Neuropathology Psychopathology Standard MRI contradictions	Neurological response during an 1-min orosensory paradigm, after a 4-h fast
Murdaugh et al., 2012 [46] Prospective observation	25 OB, scanned prior and after a 12-week dietary intervention, and on 9-month follow up	Maintenance of weight loss achieved through a 3-month behavioural intervention, 9 months post intervention	fMRI	Left-handedness IQ < 80 Chronic conditions Neuropathology Psychopathology Standard MRI contradictions	Visual stimuli of high-quality color food or non-food photographs
Weygandt et al., 2015 Prospective observation	23 OW and OB, scanned after a 12-week dietary intervention, and on 12-month follow up	Maintenance of weight loss achieved through the dietary intervention	fMRI	Psychopathology Neuropathology	Food related delay-discounting task
Simon et al., 2018 [47] Cross-sectional crossover	17 Maintainers 16 Regainers	Maintenance of weight loss ≥10% of initial body weight, 6 months after a dietary intervention	fMRI	Medication Left-handedness Psychopathology Standard MRI contradictions	Neural processing during two types of incentive delay tasks, during the anticipation and receipt of monetary and/or food-related reward

OW, Overweight; OB, Obese; NW, Normal-Weight; PET, Positron Emission Tomography; MRI, Magnetic Resonance Imaging; fMRI, functional Magnetic Resonance Imaging; BMI: Body Mass Index; IQ: Intelligence quotient.

## References

[1] Ndumele C.E., Matsushita K., Lazo M., Bello N., Blumenthal R.S., Gerstenblith G., Nambi V., Ballantyne C.M., Solomon S.D., Selvin E. (2016). Obesity and subtypes of incident cardiovascular disease. J. Am. Heart. Assoc..

[2] Langenberg C., Sharp S.J., Schulze M.B., Rolandsson O., Overvad K., Forouhi N.G., Spranger J., Drogan D., Huerta J.M., Arriola L. (2012). Long-term risk of incident type 2 diabetes and measures of overall and regional obesity: The epic-interact case-cohort study. PLoS Med..

[3] Kyrgiou M., Kalliala I., Markozannes G., Gunter M.J., Paraskevaidis E., Gabra H., Martin-Hirsch P., Tsilidis K.K. (2017). Adiposity and cancer at major anatomical sites: Umbrella review of the literature. BMJ.

[4] Monda V., La Marra M., Perrella R., Caviglia G., Iavarone A., Chieffi S., Messina G., Carotenuto M., Monda M., Messina A. (2017). Obesity and brain illness: From cognitive and psychological evidences to obesity paradox. Diabetes Metab. Syndr. Obes..

[5] Sone H., Mizuno S., Yamada N. (2005). Vascular risk factors and diabetic neuropathy. N. Engl. J. Med..

[6] Prickett C., Brennan L., Stolwyk R. (2015). Examining the relationship between obesity and cognitive function: A systematic literature review. Obes. Res. Clin. Pract..

[7] Hazar N., Seddigh L., Rampisheh Z., Nojomi M. (2016). Population attributable fraction of modifiable risk factors for Alzheimer disease: A systematic review of systematic reviews. Iran. J. Neurol..

[8] Albanese E., Launer L.J., Egger M., Prince M.J., Giannakopoulos P., Wolters F.J., Egan K. (2017). Body mass index in midlife and dementia: Systematic review and meta-regression analysis of 589,649 men and women followed in longitudinal studies. Alzheimers Dement. (Amst.).

[9] Pedditzi E., Peters R., Beckett N. (2016). Corrigenda: Corrigendum to the risk of overweight/obesity in mid-life and late life for the development of dementia: A systematic review and meta-analysis of longitudinal studies’. Age Ageing.

[10] Behary P., Miras A.D. (2014). Brain responses to food and weight loss. Exp. Physiol..

[11] Williams L.M. (2012). Hypothalamic dysfunction in obesity. Proc. Nutr. Soc..

[12] Cherbuin N., Sargent-Cox K., Fraser M., Sachdev P., Anstey K.J. (2015). Being overweight is associated with hippocampal atrophy: The path through life study. Int. J. Obes. (Lond.).

[13] Gunstad J., Paul R.H., Cohen R.A., Tate D.F., Spitznagel M.B., Grieve S., Gordon E. (2008). Relationship between body mass index and brain volume in healthy adults. Int. J. Neurosci..

[14] Gupta A., Mayer E.A., Labus J.S., Bhatt R.R., Ju T., Love A., Bal A., Tillisch K., Naliboff B., Sanmiguel C.P. (2018). Sex commonalities and differences in obesity-related alterations in intrinsic brain activity and connectivity. Obesity (Silver Spring).

[15] Morris M.J., Beilharz J.E., Maniam J., Reichelt A.C., Westbrook R.F. (2015). Why is obesity such a problem in the 21st century? The intersection of palatable food, cues and reward pathways, stress, and cognition. Neurosci. Biobehav. Rev..

[16] Jokela M., Hintsanen M., Hakulinen C., Batty G.D., Nabi H., Singh-Manoux A., Kivimaki M. (2013). Association of personality with the development and persistence of obesity: A meta-analysis based on individual-participant data. Obes. Rev..

[17] Horstmann A. (2017). It wasn’t me; it was my brain-obesity-associated characteristics of brain circuits governing decision-making. Physiol. Behav..

[18] Veronese N., Facchini S., Stubbs B., Luchini C., Solmi M., Manzato E., Sergi G., Maggi S., Cosco T., Fontana L. (2017). Weight loss is associated with improvements in cognitive function among overweight and obese people: A systematic review and meta-analysis. Neurosci. Biobehav. Rev..

[19] Napoli N., Shah K., Waters D.L., Sinacore D.R., Qualls C., Villareal D.T. (2014). Effect of weight loss, exercise, or both on cognition and quality of life in obese older adults. Am. J. Clin. Nutr..

[20] Bruce A.S., Bruce J.M., Ness A.R., Lepping R.J., Malley S., Hancock L., Powell J., Patrician T.M., Breslin F.J., Martin L.E. (2014). A comparison of functional brain changes associated with surgical versus behavioral weight loss. Obesity (Silver Spring).

[21] Montesi L., El Ghoch M., Brodosi L., Calugi S., Marchesini G., Dalle Grave R. (2016). Long-term weight loss maintenance for obesity: A multidisciplinary approach. Diabetes Metab. Syndr. Obes..

[22] Franz M.J., VanWormer J.J., Crain A.L., Boucher J.L., Histon T., Caplan W., Bowman J.D., Pronk N.P. (2007). Weight-loss outcomes: A systematic review and meta-analysis of weight-loss clinical trials with a minimum 1-year follow-up. J. Am. Diet. Assoc..

[23] Dombrowski S.U., Knittle K., Avenell A., Araujo-Soares V., Sniehotta F.F. (2014). Long term maintenance of weight loss with non-surgical interventions in obese adults: Systematic review and meta-analyses of randomised controlled trials. BMJ.

[24] Klem M.L., Wing R.R., McGuire M.T., Seagle H.M., Hill J.O. (1997). A descriptive study of individuals successful at long-term maintenance of substantial weight loss. Am. J. Clin. Nutr..

[25] Santos I., Vieira P.N., Silva M.N., Sardinha L.B., Teixeira P.J. (2017). Weight control behaviors of highly successful weight loss maintainers: The portuguese weight control registry. J. Behav. Med..

[26] Soini S., Mustajoki P., Eriksson J.G. (2016). Weight loss methods and changes in eating habits among successful weight losers. Ann. Med..

[27] Karfopoulou E., Anastasiou C.A., Hill J.O., Yannakoulia M. (2014). The medweight study: Design and preliminary results. Mediterr. J. Nutr. Metab..

[28] Shick S.M., Wing R.R., Klem M.L., McGuire M.T., Hill J.O., Seagle H. (1998). Persons successful at long-term weight loss and maintenance continue to consume a low-energy, low-fat diet. J. Am. Diet. Assoc..

[29] Brikou D., Zannidi D., Karfopoulou E., Anastasiou C.A., Yannakoulia M. (2016). Breakfast consumption and weight-loss maintenance: Results from the medweight study. Br. J. Nutr..

[30] Wyatt H.R., Grunwald G.K., Mosca C.L., Klem M.L., Wing R.R., Hill J.O. (2002). Long-term weight loss and breakfast in subjects in the national weight control registry. Obes. Res..

[31] Karfopoulou E., Brikou D., Mamalaki E., Bersimis F., Anastasiou C.A., Hill J.O., Yannakoulia M. (2017). Dietary patterns in weight loss maintenance: Results from the medweight study. Eur. J. Nutr..

[32] Catenacci V.A., Ogden L.G., Stuht J., Phelan S., Wing R.R., Hill J.O., Wyatt H.R. (2008). Physical activity patterns in the national weight control registry. Obesity (Silver Spring).

[33] Catenacci V.A., Odgen L., Phelan S., Thomas J.G., Hill J., Wing R.R., Wyatt H. (2014). Dietary habits and weight maintenance success in high versus low exercisers in the national weight control registry. J. Phys. Act. Health.

[34] Phelan S., Wyatt H.R., Hill J.O., Wing R.R. (2006). Are the eating and exercise habits of successful weight losers changing?. Obesity (Silver Spring).

[35] Yannakoulia M., Anastasiou C.A., Karfopoulou E., Pehlivanidis A., Panagiotakos D.B., Vgontzas A. (2017). Sleep quality is associated with weight loss maintenance status: The medweight study. Sleep Med..

[36] Ross K.M., Graham Thomas J., Wing R.R. (2016). Successful weight loss maintenance associated with morning chronotype and better sleep quality. J. Behav. Med..

[37] Klem M.L., Wing R.R., McGuire M.T., Seagle H.M., Hill J.O. (1998). Psychological symptoms in individuals successful at long-term maintenance of weight loss. Health Psychol..

[38] Karfopoulou E., Anastasiou C.A., Avgeraki E., Kosmidis M.H., Yannakoulia M. (2016). The role of social support in weight loss maintenance: Results from the medweight study. J. Behav. Med..

[39] Anastasiou C.A., Fappa E., Karfopoulou E., Gkza A., Yannakoulia M. (2015). Weight loss maintenance in relation to locus of control: The medweight study. Behav. Res. Ther..

[40] Teixeira P.J., Silva M.N., Coutinho S.R., Palmeira A.L., Mata J., Vieira P.N., Carraca E.V., Santos T.C., Sardinha L.B. (2010). Mediators of weight loss and weight loss maintenance in middle-aged women. Obesity (Silver Spring).

[41] DelParigi A., Chen K., Salbe A.D., Hill J.O., Wing R.R., Reiman E.M., Tataranni P.A. (2004). Persistence of abnormal neural responses to a meal in postobese individuals. Int. J. Obes. Relat. Metab. Disord..

[42] DelParigi A., Chen K., Salbe A.D., Hill J.O., Wing R.R., Reiman E.M., Tataranni P.A. (2007). Successful dieters have increased neural activity in cortical areas involved in the control of behavior. Int. J. Obes. (Lond.).

[43] McCaffery J.M., Haley A.P., Sweet L.H., Phelan S., Raynor H.A., Del Parigi A., Cohen R., Wing R.R. (2009). Differential functional magnetic resonance imaging response to food pictures in successful weight–loss maintainers relative to normal–weight and obese controls. Am. J. Clin. Nutr..

[44] Hassenstab J.J., Sweet L.H., Del Parigi A., McCaffery J.M., Haley A.P., Demos K.E., Cohen R.A., Wing R.R. (2012). Cortical thickness of the cognitive control network in obesity and successful weight loss maintenance: A preliminary mri study. Psychiatry Res..

[45] Sweet L.H., Hassenstab J.J., McCaffery J.M., Raynor H.A., Bond D.S., Demos K.E., Haley A.P., Cohen R.A., Del Parigi A., Wing R.R. (2012). Brain response to food stimulation in obese, normal weight, and successful weight loss maintainers. Obesity (Silver Spring).

[46] Murdaugh D.L., Cox J.E., Cook E.W., Weller R.E. (2012). Fmri reactivity to high–calorie food pictures predicts short-and long-term outcome in a weight-loss program. Neuroimage.

[47] Simon J.J., Becker A., Sinno M.H., Skunde M., Bendszus M., Preissl H., Enck P., Herzog W., Friederich H.C. (2018). Neural food reward processing in successful and unsuccessful weight maintenance. Obesity (Silver Spring).

[48] Perri M.G., Nezu A.M., McKelvey W.F., Shermer R.L., Renjilian D.A., Viegener B.J. (2001). Relapse prevention training and problem–solving therapy in the long–term management of obesity. J. Consult. Clin. Psychol..

[49] Volkow N.D., Wang G.J., Baler R.D. (2011). Reward, dopamine and the control of food intake: Implications for obesity. Trends Cogn. Sci..

[50] Pursey K.M., Stanwell P., Callister R.J., Brain K., Collins C.E., Burrows T.L. (2014). Neural responses to visual food cues according to weight status: A systematic review of functional magnetic resonance imaging studies. Front. Nutr..

[51] Goldman R.L., Canterberry M., Borckardt J.J., Madan A., Byrne T.K., George M.S., O’Neil P.M., Hanlon C.A. (2013). Executive control circuitry differentiates degree of success in weight loss following gastric-bypass surgery. Obesity (Silver Spring).

[52] Bruce J.M., Hancock L., Bruce A., Lepping R.J., Martin L., Lundgren J.D., Malley S., Holsen L.M., Savage C.R. (2012). Changes in brain activation to food pictures after adjustable gastric banding. Surg. Obes. Relat. Dis..

[53] Le D.S., Pannacciulli N., Chen K., Salbe A.D., Del Parigi A., Hill J.O., Wing R.R., Reiman E.M., Krakoff J. (2007). Less activation in the left dorsolateral prefrontal cortex in the reanalysis of the response to a meal in obese than in lean women and its association with successful weight loss. Am. J. Clin. Nutr..

[54] Le D.S., Pannacciulli N., Chen K., Del Parigi A., Salbe A.D., Reiman E.M., Krakoff J. (2006). Less activation of the left dorsolateral prefrontal cortex in response to a meal: A feature of obesity. Am. J. Clin. Nutr..

[55] Weygandt M., Mai K., Dommes E., Ritter K., Leupelt V., Spranger J., Haynes J.D. (2015). Impulse control in the dorsolateral prefrontal cortex counteracts post-diet weight regain in obesity. Neuroimage.

[56] Val-Laillet D., Aarts E., Weber B., Ferrari M., Quaresima V., Stoeckel L.E., Alonso-Alonso M., Audette M., Malbert C.H., Stice E. (2015). Neuroimaging and neuromodulation approaches to study eating behavior and prevent and treat eating disorders and obesity. Neuroimage Clin..

[57] Lezak M.D., Howieson D.B., Loring D.W., Hannay H.J., Fischer J.S. (2012). Neuropsychological Assessment.

[58] Nederkoorn C., Houben K., Hofmann W., Roefs A., Jansen A. (2010). Control yourself or just eat what you like? Weight gain over a year is predicted by an interactive effect of response inhibition and implicit preference for snack foods. Health Psychol..

[59] Spitznagel M.B., Alosco M., Strain G., Devlin M., Cohen R., Paul R., Crosby R.D., Mitchell J.E., Gunstad J. (2013). Cognitive function predicts 24-month weight loss success following bariatric surgery. Surg. Obes. Relat. Dis..

[60] Gettens K.M., Gorin A.A. (2017). Executive function in weight loss and weight loss maintenance: A conceptual review and novel neuropsychological model of weight control. J. Behav. Med..

[61] Jones A., Hardman C.A., Lawrence N., Field M. (2018). Cognitive training as a potential treatment for overweight and obesity: A critical review of the evidence. Appetite.

[62] Dalle Grave R., Sartirana M., El Ghoch M., Calugi S. (2017). Personalized multistep cognitive behavioral therapy for obesity. Diabetes Metab. Syndr. Obes..

[63] Zhao J., Li M., Zhang Y., Song H., Von Deneen K.M., Shi Y., Liu Y., He D. (2017). Intrinsic brain subsystem associated with dietary restraint, disinhibition and hunger: An fmri study. Brain Imaging Behav..

[64] Olivo G., Zhou W., Sundbom M., Zhukovsky C., Hogenkamp P., Nikontovic L., Stark J., Wiemerslage L., Larsson E.-M., Benedict C. (2017). Resting-state brain connectivity changes in obese women after roux-en-y gastric bypass surgery: A longitudinal study. Sci. Rep..

[65] Pandit R., Mercer J.G., Overduin J., La Fleur S.E., Adan R.A. (2012). Dietary factors affect food reward and motivation to eat. Obes. Facts.

[66] Dalle Grave R., Calugi S., El Ghoch M. (2018). Are personality characteristics as measured by the temperament and character inventory (TCI) associated with obesity treatment outcomes? A systematic review. Curr. Obes. Rep..

[67] Monda V., Salerno M., Sessa F., Bernardini R., Valenzano A., Marsala G., Zammit C., Avola R., Carotenuto M., Messina G. (2018). Functional changes of orexinergic reaction to psychoactive substances. Mol. Neurobiol..

[68] Chieffi S., Carotenuto M., Monda V., Valenzano A., Villano I., Precenzano F., Tafuri D., Salerno M., Filippi N., Nuccio F. (2017). Orexin system: The key for a healthy life. Front. Physiol..

[69] Boughton C.K., Murphy K.G. (2013). Can neuropeptides treat obesity? A review of neuropeptides and their potential role in the treatment of obesity. Br. J. Pharmacol..

[70] Jensen M.D., Ryan D.H., Apovian C.M., Ard J.D., Comuzzie A.G., Donato K.A., Hu F.B., Hubbard V.S., Jakicic J.M., Kushner R.F. (2014). 2013 AHA/ACC/TOS guideline for the management of overweight and obesity in adults: A report of the American college of cardiology/American heart association task force on practice guidelines and the obesity society. Circulation.

[71] Hinkle W., Cordell M., Leibel R., Rosenbaum M., Hirsch J. (2013). Effects of reduced weight maintenance and leptin repletion on functional connectivity of the hypothalamus in obese humans. PLoS ONE.

[72] Greenway F.L. (2015). Physiological adaptations to weight loss and factors favouring weight regain. Int. J. Obes. (Lond.).

[73] Huang Y.-T., Maccani J.Z.J., Hawley N.L., Wing R.R., Kelsey K.T., McCaffery J.M. (2015). Epigenetic patterns in successful weight loss maintainers: A pilot study. Int. J. Obes. (Lond.).

